# *CLOCK* Polymorphisms in Attention-Deficit/Hyperactivity Disorder (ADHD): Further Evidence Linking Sleep and Circadian Disturbances and ADHD

**DOI:** 10.3390/genes10020088

**Published:** 2019-01-28

**Authors:** Marina Xavier Carpena, Mara H. Hutz, Angélica Salatino-Oliveira, Guilherme V. Polanczyk, Cristian Zeni, Marcelo Schmitz, Rodrigo Chazan, Julia P. Genro, Luis Augusto Rohde, Luciana Tovo-Rodrigues

**Affiliations:** 1 Federal University of Pelotas, Post-Graduate Program in Epidemiology, Pelotas 96020-220, Brazil; marinacarpena_@hotmail.com; 2 Federal University of Rio Grande do Sul, Department of Genetics, Porto Alegre 915010-970, Brazil; mara.hutz@ufrgs.br (M.H.H.); geli.oliveira@gmail.com (A.S.-O.); 3 Universidade de Sao Paulo, Faculdade de Medicina FMUSP, Department of Psychiatry, Sao Paulo 05403-903, Brasil; gvp@usp.br; 4 University of Texas Health Sciences Center at Houston, Houston, TX 75457, USA; cpzeni@gmail.com; 5 Federal University of Rio Grande do Sul, Department of Psychiatry, Child & Adolescent Psychiatry Unit, Hospital de Clinicas de Porto Alegre, Porto Alegre 90035-007, Brazil; maschmitz@hcpa.edu.br (M.S.); lrohde@terra.com.br (L.A.R.); 6 Federal University of Rio Grande do Sul, Post-graduate Program in Psychiatry and Behavioral Sciences, Porto Alegre 90035-007, Brazil; rchazan@yahoo.com; 7 Programa de Pós-Graduação em Biociências, Universidade Federal de Ciências da Saúde de Porto Alegre, Porto Alegre 90050-170, Brazil; juliagenro@hotmail.com

**Keywords:** *CLOCK* gene, ADHD, circadian rhythm, sleep problem, inattention symptoms, genetic, complex diseases, Latin America

## Abstract

Circadian and sleep disorders, short sleep duration, and evening chronotype are often present in attention-deficit/hyperactivity disorder (ADHD). *CLOCK*, considered the master gene in the circadian rhythm, has been explored by few studies. Understanding the relationship between ADHD and *CLOCK* may provide additional information to understand the correlation between ADHD and sleep problems. In this study, we aimed to explore the association between ADHD and *CLOCK*, using several genetic markers to comprehensively cover the gene extension. A total of 259 ADHD children and their parents from a Brazilian clinical sample were genotyped for eight single nucleotide polymorphisms (SNPs) in the *CLOCK* locus. We tested the individual markers and the haplotype effects using binary logistic regression. Binary logistic and linear regressions considering ADHD symptoms among ADHD cases were conducted as secondary analysis. As main result, the analysis showed a risk effect of the G-A-T-G-G-C-G-A (rs534654, rs1801260, rs6855837, rs34897046, rs11931061, rs3817444, rs4864548, rs726967) haplotype on ADHD. A suggestive association between ADHD and rs534654 was observed. The results suggest that the genetic susceptibility to circadian rhythm attributed to the *CLOCK* gene may play an important role on ADHD.

## 1. Introduction

Attention-deficit/hyperactivity disorder (ADHD) is one of the most common neurodevelopment disorders during childhood and adolescence, affecting ~5% of people at this age worldwide [1]. ADHD core symptoms, characterized by inattention, hyperactivity, and impulsivity, are associated with cognitive impairments. A high prevalence of comorbidities is reported, making ADHD a complex and heterogeneous phenotype [2]. Sleep/circadian rhythm problems are among the medical conditions associated with ADHD that have recently received attention. Individuals affected by ADHD more often present with circadian and sleep disorders, are short sleepers, and frequently present evening chronotype [3,4,5]. For example, 55–75% of parents reported sleep quality changes in their children with ADHD [6,7,8].

The circadian rhythm comprises a regulation underlying a 24 hour-physiological cycle, including metabolism, body temperature, hormone secretion, and sleep/wake patterns in mammals, and it is especially important for sleep behavior [9,10]. It is also controlled by a complex system of molecular regulation with a master precursor, located in the suprachiasmatic nucleus (NSQ) of the anterior hypothalamus. *CLOCK* is one of the most important genes of the endogenous master clock system. The main function of this gene relies on the transcription activation of downstream core clock genes and on the promotion of rhythmic chromatin opening, also regulating DNA accessibility of other transcription factors [11]. In humans, the *CLOCK* gene has already been associated with the evening chronotype as well as with some circadian and sleep disorders, such as delayed sleep phase syndrome [11].

Animal experiments concerning the *CLOCK* gene provide putative mechanistic links between circadian/sleep disorder and ADHD pathophysiology [12,13,14,15]. An important role of *CLOCK* in neuronal function, mainly in dopamine output regulation, has been demonstrated [13,16]. Furthermore, methylphenidate and atomoxetine, two drugs efficacious to treat ADHD, induced modification in *CLOCK* as well as in other circadian genes’ expression, providing additional evidence linking circadian system regulation and ADHD [12,14,15]. 

The nature of the association between sleep and circadian problems and ADHD is unclear [3]. An understanding of the role of relevant molecular mechanisms for the association between both phenotypes may provide important information to predict ADHD or sleep problems. Few candidate gene studies in humans have explored such mechanisms, and the *CLOCK* gene has been the most investigated [11,17,18,19]. All studies investigating the association between the *CLOCK* gene and ADHD focused only on one genetic variant: The 3’UTR rs1801260 SNP. Only one study has evaluated several variants and observed a haplotype effect [17]. A risk effect of rs1801260 T allele on the ADHD phenotype was consistent among these studies [11,17,18,19]. 

The association studies between *CLOCK* and ADHD described above used European and/or Asian samples. Population genetic structure, allele frequency, and heritability could vary across populations around the world [20], stressing the need for replication studies in order to clarify gene function in the phenotype. For instance, in other psychiatric disorders, where the *CLOCK* gene is more explored, ancestry seems to be an important factor in understanding the mixed results. The association direction and magnitude seem to be highly impacted considering ancestry [11]. To the best of our knowledge, the association between the *CLOCK* gene and ADHD has never been explored in admixed populations, especially from Latin America. Therefore, we aimed to explore the association between the *CLOCK* gene and ADHD, using several genetic markers to comprehensively cover the gene extension in Brazilian patients with ADHD. 

## 2. Material and Methods

### 2.1. Sample

A sample of 259 Brazilian probands with ADHD and their parents were enrolled in this study. The probands were recruited at the ADHD Outpatient Program (ProDAH) from Hospital de Clínicas de Porto Alegre (HCPA). ADHD was diagnosed according to the Diagnostic and Statistical Manual of Mental Disorders (DSM-IV) criteria [21]. The assessment process followed a previously reported three-stage protocol [22], including the application of semi-structured diagnostic interviews (Schedule for Affective Disorders and Schizophrenia for School Age Children Present and Lifetime Version- KSADS-PL) by trained research assistants, and clinical assessments by experienced child psychiatrists. The Swanson, Nolan, and Pelham Scale-Version IV (SNAP-IV) was rated by child psychiatrists blinded to genotype to assess symptom severity. This scale is made up of nine items both in the inattention and hyperactive/impulsivity symptom domains and the wording is based on DSM-IV. Each SNAP-IV item is rated on a scale from absence of (score = 0) to severe symptoms (score = 3). This scale has been frequently used [22,23,24] and recently validated and is considered as a reliable scale in a Brazilian sample [25]. 

### 2.2. DNA and Genotyping

Blood samples were collected from probands and their parents. DNA was extracted from lymphocytes by standard procedures [26]. DNA from all samples was quantified by spectrophotometry using NanoDrop 1000 (Thermo Fisher Scientific Inc., Waltham, MA, USA). Infinium PsychArray-24 BeadChip, an array developed for studies focused on psychiatric predisposition and risk, was used to genotype both children and parents, in a total of ~593,260 markers. Quality control and imputation were done using the Rapid Imputation Consortium Pipeline (Ricopili), a pipeline developed and used by the Psychiatric Genome Consortium (PGC, available at https://github.com/Nealelab/ricopili), which covers both individual and markers quality control (QC). The filters used before imputation were Hardy–Weinberg equilibrium (HWE)-*p*-value < 1 × 10^−6^ calculated from founders and exclusion of invariant variants. 

The Brazilian sample comes from a population of essentially European ancestry with little evidence of African or Native American admixture. The Principal Component Analysis considering genotyped markers shows an overlap between the Brazilian samples and the European population from 1000 Genomes Project (Figure S1). Additional markers were imputed using 1000 Genomes Project European population phase 3 as a reference panel. After imputation, we obtained a total of 11,799,192 variants.

For the secondary analysis considering symptoms’ severity (see Section 2.5 Secondary Analyses), an adjustment for the Principal Components to control for population stratification was done. The Principal Components Analysis was carried out considering all genotyped and imputed markers using the following filters: Missing rate of 0.1, a quality INFO higher than 0.8, and HWE *p*-value higher than 1 × 10^−6^.

### 2.3. Genetic Markers

In order to examine the *CLOCK* gene extension, markers included in the Infinium PsychArray-24 BeadChip inside the *CLOCK* gene and those 10 kb upstream and downstream the gene with valid genotype in more than 90% of the sample were included in the analysis. In total, eight genotyped single nucleotide polymorphisms (SNPs) were included.

### 2.4. Statistical Analysis

For the 259 trio data, we applied a case-pseudo control design, which is an alternative procedure to the genotype transmission/disequilibrium test in which the pseudo control is created with the non-transmitted alleles from the parents [27]. Allele and genotypic frequencies were estimated by counting in children. Hardy-Weinberg was estimated by Χ^2^ test, implemented in PLINK 1.9 software [28,29]. 

In order to estimate the effect of individual markers, binary logistic regression was performed using an additive genetic model. The estimates are given through the odds ratio (OR), 95% confidence interval, and *p*-value. These analyses were performed using PLINK 1.9 [28]. The *p*-value for multiple tests was estimated by Type 1 Error Calculator GEC [30], a Java-based application developed to address multiple-testing issue with dependent SNPs. Based on linkage disequilibrium, it provides the independent number of tests or redundancy degree in a set of dependent SNPs as well as the *p*-value thresholds to declare significant SNPs for association in a set of dependent *p*-values. For our set of markers, a total of six effective markers was detected. According to GEC, the *p*-value threshold for significance was set at 0.0081 and, suggestive, at 0.0162. Since pseudo controls are perfectly matched to each case, no covariates were used in the association analysis.

Linkage disequilibrium and visualization were done by Haploview [31]. Haplotype analyses were performed using UNPHASED 3.1.7 software [32]. The global and individual tests for haplotypes are reported. The first is a global, or omnibus, test of whether any haplotypes are associated, estimated by a likelihood ratio test. The *p*-value is the probability of observing a likelihood ratio statistic at least as large as this one, if the null hypothesis was true. Then, the estimated haplotype effects against the reference are reported by OR and their 95% confidence interval. For the haplotype analyses only, haplotypes with a frequency higher that 0.01 were considered.

### 2.5. Secondary Analyses

As a secondary analysis, we explored the symptomatology of hyperactivity/impulsivity, inattention and total ADHD in patients (obtained from KSADS-PL). Symptom data was available for 221 subjects. To analyze this data, two regression models were considered: Binary logistic and linear regression. First, the symptoms were categorized using the percentile 50%. Since we identified that from this percentile, the symptom number showed a greater difference, we were able to split the sample into higher and lower scores (Table S1). The cut-off used for inattention was 9, for hyperactivity/impulsivity, 8, and for global ADHD, 14 symptoms. Alternatively, a linear regression was performed using the number of symptoms as the outcome. These analyses were adjusted for sex, age, and genomic ancestry, using the first five principal components described above.

### 2.6. In Silico Functionality Analysis

In order to provide further information about the putative functionality of each marker included in this study, we used the regulatory and functional information provided by Regulome DB, HaploReg v4.1 [33], Varian Effect Predictor (VEP) [34] and Combined Annotation Dependent Depletion (CADD) [35]. The assembly GRCh37 and RefSeq NM_004898.3 were used as a reference. HaploReg [33] is a tool for exploring annotations of the noncoding genome in variants. Information on the chromatin state and protein binding annotation from the Roadmap Epigenomics and ENCODE projects are functional evidence integrated in this dataset. RegulomeDB [36] is a database of SNPs with known and predicted regulatory elements integrating data from GEO, the ENCODE project, and published literature in the intergenic regions of the *H. sapiens* genome. It attributes a score for functionality, which varies from 1 to 6 and the lower the value, the higher the probability of impact in a regulatory site. VEP determines the effect of variants (SNPs, insertions, deletions, CNVs, or structural variants) on genes, transcripts, and protein sequences, as well as regulatory regions. The CADD v1.4 provides a measure of variant deleteriousness namely the normalized score, PHRED-scaled, with higher values indicating that a variant is more likely to have deleterious effects [35].

### 2.7. Research Ethics

This study was approved by the Ethics Committee of Hospital de Clínicas de Porto Alegre (HCPA) (Project identification code 45210715.1.0000.5327) and was conducted in accordance with the Declaration of Helsinki. All subjects gave their informed consent for inclusion before they participated in the study. The parents provided written informed consent and probands provided verbal assent to participate.

## 3. Results

Table 1 shows the demographic and health characteristics of the probands included in the study. Most children were boys (76.4%), almost half were diagnosed with ADHD combined subtype (47.1%), and 83.4% had white skin color. The mean age was 10.42 years (range: 4–17).

The results for the association analysis considering the eight markers included in the study are shown in Table 2. None of the markers reached the significance level considering multiple tests correction. A suggestive association emerged from rs534654, with the A as the risk allele (OR = 1.54; IC95% = 1.11–2.12). 

**Figure 1:**
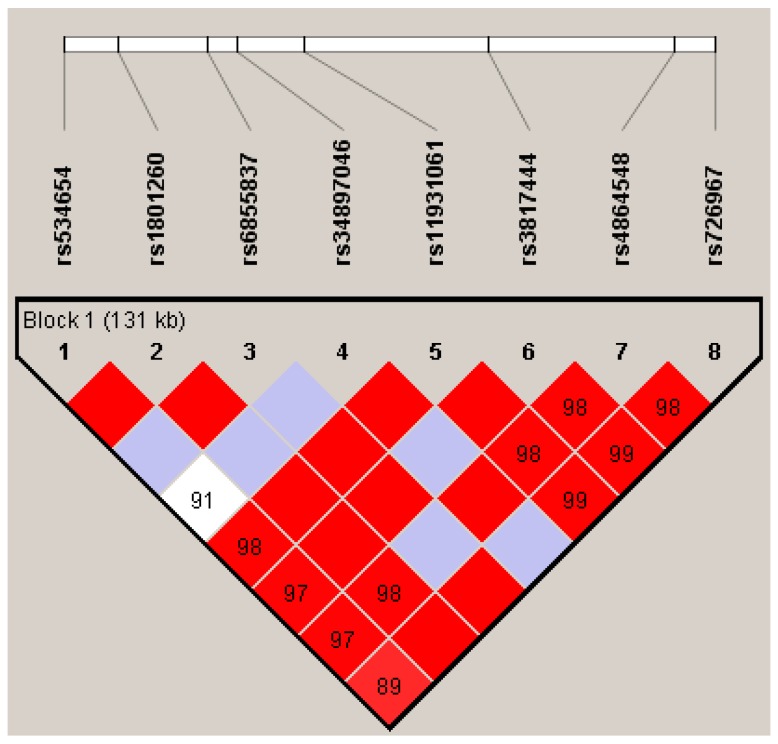
Linkage disequilibrium (LD) for the *CLOCK* locus for familial data. The standard Haploview LD color scheme based on D’ and log of the likelihood odds ratio (LOD) is used and the value of 100 × D’ for each SNP pair is given in its respective tile unless D’ = 1. Color scheme: bright red: D’ = 1, LOD ≥ 2; blue: D’ = 1, LOD < 2, shades of pink/red: D’ < 1, LOD ≥ 2; white: D’ < 1, LOD < 2.

Figure 1 shows the linkage disequilibrium pattern in the *CLOCK* locus. High pairwise D’ values were observed, and all SNPs were in a single block. The global association test suggested a significant effect for at least one haplotype (*p* = 0.027). The individual haplotype effect showed that G-A-T-G-G-C-G-A was considered as a risk genetic factor for ADHD (OR = 2.15, IC95% = 1.04; 4.43) (Table 3). 

### 3.1. Secondary Analysis

The logistic regression model for symptomatology showed that three SNPs were nominally associated with inattention symptoms: rs11931061 (risk allele G; OR = 1.561; CI95%: 1.046; 2.329), rs3817444 (risk allele A; OR = 1.60; CI95%: 1.06; 2.42), and rs726967 (risk allele T; OR = 1.57; CI95%: 1.03; 2.37) (Table 4). The last two were also marginally associated when linear regression was performed (Table S3). However, in general, the results were not consistent between both regression models.

### 3.2. In Silico Functionality Analysis

In silico analyses were performed to predict the functionality of the *CLOCK* variants to better explore our results. According to HaploReg and RegulomeDB data, rs4864548 and rs1801260 are placed in regulatory sites (Table S4). rs4864548 is located at position 727 upstream of the gene. It was observed to be placed in the region of promoter marks in 23 tissues and enhancer marks in two tissues. It is also considered an expression quantitative trait loci (eQTL) for the *CLOCK* gene in several tissues (esophagus mucosa, lung, tibial nerve, skin, and thyroid). rs1801260 is a 3’ UTR variant placed in a site of interaction with Transcriptional repressor CTCF (CTCF), Basic leucine zipper transcriptional factor ATF-like (BATF), and Signal transducer and activator of transcription 3 (STAT3) proteins. It is also an eQTL for *CLOCK*.

Considering the regulatory regions specific in brain tissue, both markers are placed in active domains of chromatin (Table S5). The active site (based on the Roadmap Epigenomics Consortium 25-state mode) for rs4864548 is 2_PromU, which is present in all the available brain regions and developmental stages, and, for rs1801260, it is 11_TxEnh3, which is present only in adult brain regions. 

According to CADD, the highest scores are observed for the coding variants (rs6855837, placed in exon 15, and rs34897046, placed in exon 10; Table S4). Both are missense variants. However, there was no strong suggestion of a damaging effect on protein by both predictions using PolyPhen and SIFT included in VEP.

Considering the rs534654 variant, histone promoter marks were identified in brain tissue; However, it has low probability of affecting regulatory function according to RegulomeDB and has low scores in CADD. Elusive functional regulatory function was observed for rs3817444 and rs726967, for which histone marks were observed in other tissues except the brain. The rs11931061 is placed in an intronic region and no potential functional impact can be inferred for this marker according to the tools used in this study (Tables S4 and S5).

## 4. Discussion

In this study, we tested the association between the *CLOCK* gene and ADHD using family data. We included several genetic markers to comprehensively cover the gene extension. Our results point toward a haplotype risk effect of the gene on ADHD. Considering individual markers, a suggestive role of rs534654 on ADHD susceptibility was observed.

Several lines of evidence link the *CLOCK* gene with ADHD. First, the *CLOCK* gene has an important function in dopaminergic regulation as well as in neurocognitive function, both pathways linking wake/sleep patterns to ADHD etiology [13,16]. Second, the expression of genes involved in neuronal migration is regulated by *CLOCK*, and its dysregulation was reported to disrupt coexpressed gene networks implicated in neuropsychiatric disorders [37]. The literature has also reported that the circadian rhythm may play an essential role on the effect of ADHD treatment medication [4,38]. Finally, in humans, an association between the *CLOCK* gene and psychiatric disorders has already been reported [3,5]. For ADHD, only three studies showed an association of the rs1801260 T allele with ADHD [17,18,19].

We covered markers in a block of 131kb in the *CLOCK* gene loci, showing a high pairwise linkage disequilibrium. Our haplotypic analysis suggests that the *CLOCK* gene may play a role on ADHD. The haplotype, G-A-T-G-G-C-G-A (rs534654, rs1801260, rs6855837, rs34897046, rs11931061, rs3817444, rs4864548, rs726967), was associated with an increased ADHD risk. According to *in sillico* functionality analysis, many variants in this haplotype present a putative functional role, mainly rs4864548, rs1801260, rs6855837, and rs34897046. None of the eight variants was significantly associated with ADHD when analyzed separately. The only study in the literature exploring haplotypes in ADHD observed that a single haplotype block, covering 73 kb (rs3805148 (A/C), rs12504300 (C/G), rs4864542 (C/G), and rs12649507 (A/G)), was associated with inattention symptoms [17].

Although the functional molecular role in vivo and in vitro presents compelling findings linking the gene with ADHD etiology, the mechanism through which sleep/circadian rhythms problems affect ADHD still needs to be elucidated. The most consolidated theory about the association between circadian rhythm and ADHD is that sleep deprivation and circadian disorders would lead to a deficit in pre-frontal cortex executive functions, which at the pathological level, would represent ADHD symptoms [3,39,40]. Our results of the markers were not robustly and consistently associated with specific ADHD symptoms. Further studies exploring the association with *CLOCK* and other clock genes and ADHD subtypes are needed. 

The SNP rs534654 showed the strongest individual association signal in our study. However, this SNP did not survive to multiple test correction. This variant has never been tested in ADHD. It was already reported as nominally significantly associated with bipolar disorder in a genome-wide association study [41] and in a family based study [42]. It was also observed to interact with rs6442925 in the *BHLHB2* gene and rs1534891 in the *CSNK1E* gene in association with bipolar disorder. 

Considering the markers with potential regulatory effects in the *CLOCK* gene, we were not able to find any study exploring the SNP rs4864548 in ADHD susceptibility. On the other hand, the studies concerning ADHD were mostly conducted with rs1801260. It is of note that we were not able to replicate the previous findings, which observed an association with rs1801260 (3111T/C). This polymorphism is located in an interaction site with miRNA-182 [43,44], an important region for mRNA stability, expression, and function [11]. Increasing evidence has shown that miRNA-182 may play a role in the regulation of synaptic protein synthesis in long-lasting plasticity, modulating synaptic plasticity [45], as well as maintaining neural physiological function and maturation [46,47]. Furthermore, miRNA-182 was associated with some neurological and psychiatric phenotypes, such as depression [46,47,48,49].

Kissling et al. (2008) and Xu et al. (2010) found a risk effect of the T allele of rs1801260 on ADHD in a clinical European adult sample and a clinical European and Asian child sample, respectively. Jeong et al.(2014) also observed that rs1801260 (T allele) was associated with ADHD scores in an Korean adult sample [17]. These three studies are all case-control designed with a gene-candidate approach. Although the direction of association in our analysis was consistent with the previous reported, we found no association between this variant and ADHD in this study. Despite the possible low power limitation, it is possible that other variants may be important in our sample, as suggested by haplotypic analysis [11]. 

Our results should be interpreted considering its limitations. The first is related to sample size. We studied a small clinical sample and our potential to find positive association was limited due to low statistical power. However, it is important to note that the sample sizes of other studies involving the *CLOCK* gene and ADHD are comparable to ours [18,19]. Second, we lacked sleep/biological rhythm information. The inclusion of sleep habits/other circadian characteristic variables would benefit the analysis. It would be possible, for example, to explore mechanisms that may explain the association, such as a mediation effect of sleep patterns. Finally, working with admixed population may incur population stratification problems [27]. However, the family-based association design of our study is robust against stratification. 

It is important to highlight that the *CLOCK* gene was neither associated with ADHD nor with chronotype, sleep duration, or sleep disorders in the most comprehensive and recent genome-wide association studies (GWAS) [50,51,52,53]. Although we cannot discard that our findings might reflect spurious associations, some issues deserve to be mentioned. First, it is considered the master gene in the circadian rhythm and its function has been well studied in animal experiments [11]. Second, the *CLOCK* gene may be a peripheric gene of small effect on ADHD, involved in the regulation of core genes, as proposed by the omnigenic model of complex traits [54]. Then, even though it may play a role in ADHD, GWASs may have small power to detect it as statistically significant. Also, considering the highly polygenic genetic architecture of ADHD, gene−gene interactions, gene−environment interactions, or gene−environment correlations may also be important to explain the ADHD heritability [7,55]. For *CLOCK*, the literature is scarce and studies aiming to explore it are needed. Additionally, it is possible that the *CLOCK* gene may be more related to a specific ADHD subtype. Then, the use of global ADHD phenotypes would mask the *CLOCK* gene effect in this disorder.

To the best of our knowledge, this is the first study to explore the relationship between several *CLOCK* gene polymorphisms and ADHD phenotypes in a non-European and non-Asian sample. Brazil is a country of continental proportions and unique populational admixture characteristics. The population has originated from a tri-ethnic parental populations (Native Americans, Africans, and Europeans), which resulted in one of the most admixed and heterogeneous populations in the world [56]. Within a country of continental size, like Brazil, population composition varies widely among regions, as may be generally expected from Brazilian history. More European influence is observed in the south, while the African contribution predominates in the northeast and the Amerindian in the north. Our sample from the south of Brazil was composed by a majority of European ancestry, presenting little evidence of African or Native American admixture [57,58,59]. Admixed populations are a challenge concerning genetic association studies. Several genetic factors, such as LD patterns and allele frequencies, may vary across populations [60], which have been previously presented for the *CLOCK* gene [11]. Furthermore, effect sizes may differ among populations, at least for some traits, and allelic heterogeneity could have an important impact on the generalizability potential of association results across populations. Failures in transferability findings for admixed Hispanics/Latinos have been clearly demonstrated for polygenic risk scores [60] Therefore, the differences between our findings to the previous ones reported may arise from that. The GWASs published to date were conducted in European ancestry population. Considering the populational and generalizability factors discussed above, one cannot exclude the possibility of *CLOCK* being important for these phenotypes in other population ancestries. 

In conclusion, we provided additional evidence suggesting a putative role of *CLOCK* and circadian rhythm/sleep problems in ADHD. It is plausible to expect that the relation between sleep/circadian rhythm phenotypes and ADHD may arise from a polygenic complex mechanism involving several clock genes as well as different SNPs, besides the regulation through the *CLOCK* gene. Further studies, especially based on genome-wide approaches, are needed to clarify the role of *CLOCK*, other clock genes, and sleep phenotypes in ADHD clinical subtypes in different populations.

## Figures and Tables

**Table 1:** Sample characterization according to demographic variables and attention-deficit/hyperactivity disorder (ADHD) presentations.

		Variable N	N (%)
			Mean (Standard Deviation, SD)
Age(years), mean (SD)		259	10.42 (3.2)
IQ, mean (SD)		255	93.52 (13.1)
Gender, N male (%)		259	198 (76.4)
Skin color		259	
White, N (%)			216(83.4)
Brown or Black, N (%)			43 (16.6)
ADHD presentations		259	
Inattentive, N (%)			114(44.0)
Hyperactive, N (%)			13 (5.0)
Combined, N (%)			122 (47.1)
Subthreshold N (%)			10 (3.9)
Comorbid Conditions			
Conduct Disorders, N (%)		258	36 (13.9)
Oppositional Defiant Disorders, N (%)		258	92 (35.5)
Mood disorders, N (%)		241	23 (8.9)
Anxiety disorders, N (%)		241	72 (27.8)

**Table 2:** Effect of allele frequency and Hardy-Weinberg deviation statistics in ADHD cases, and binary logistic regression association estimates considering ADHD cases and pseudo control design (N = 259 cases and 259 pseudo controls).

SNP	Location	Position Regarding *CLOCK* Gene	EA	RA	FREQ EA *	HWE (*p*-value)	OR (95% CI)	*p*
rs534654	56290220	3848 bp downstream	A	G	0.226	0.593	1.54 (1.11–2.12)	0.010
rs1801260	56301369	exon 23 (3’UTR)	G	A	0.253	0.252	0.87 (0.66–1.15)	0.329
rs6855837	56319244	exon 15	T	G	0.048	0.456	1.96 (0.99–3.86)	0.054
rs34897046	56325365	exon 10	C	G	0.029	0.188	0.79 (0.40–1.56)	0.492
rs11931061	56338793	intron 7	G	A	0.402	0.120	1.180 (0.92–1.51)	0.188
rs3817444	56375981	intron 2	A	C	0.344	0.075	1.059 (0.82–1.36)	0.655
rs4864548	56413803	727 bp upstream	A	G	0.344	0.681	0.911 (0.71–1.18)	0.473
rs726967	56421713	8637 pb upstream	T	A	0.334	0.094	1.068 (0.83–1.37)	0.608

* The allele frequency and HWE are presented for cases (probands) only; EA: Effect allele for the present study; RA: Reference allele for the present study; FREQ EA: Effect allele frequency; HWE: Hardy-Weinberg Equilibrium; SNP: Single nucleotide polymorphism; OR: Odds Ratio; CI: Confidence Interval; GRCh37: Genome reference assembly. Alleles considering the forward strand as reference. The allele frequency in the European, African, and American populations form 1000 Genome Project are shown in the Table S2.

**Table 3:** Haplotype association analysis considering cases and pseudo control design (N = 259 cases and 259 pseudo controls).

Haplotype *	Haplotype Frequency **	OR (95% CI)
G-A-G-G-A-C-A-A	0.314	1
G-G-G-G-A-C-G-A	0.255	0.92 (0.66–1.27)
A-A-G-G-G-A-G-T	0.213	1.43 (1.00–2.07)
G-A-G-G-G-A-G-T	0.125	0.77 (0.52–1.15)
G-A-T-G-G-C-G-A	0.050	2.15 (1.04–4.43)
G-A-G-C-A-C-A-A	0.030	0.82 (0.40–1.66)
A-A-G-G-G-A-G-A	0.012	1.24 (0.37–4.14)

* Haplotype considering markers in the following order: rs534654, rs1801260, rs6855837, rs34897046, rs11931061, rs3817444, rs4864548, rs726967. Alleles considering the forward strand as the reference; ** Haplotype frequencies for cases.

**Table 4:** Results for secondary analysis exploring the association between *CLOCK* variants and inattention, hyperactivity, and total ADHD symptoms in the ADHD probands sample (N = 221).

		Inattention		Hyperactivity		ADHD Total Symptoms	
SNP	EA	OR (95%CI95) *	*p* *	OR (95%CI95) *	*p* *	OR (95%CI95) *	*p* *
rs534654	A	1.53 (0.94–2.5)	0.088	1.157 (0.71–1.90)	0.565	1.16 (0.72; 1.88)	0.534
rs1801260	G	0.70 (0.45–1.10)	0.122	0.774 (0.49–1.22)	0.265	0.80 (0.52; 6.93)	0.309
rs6855837	T	1.51 (0.55–4.15)	0.424	2.654 (0.94–7.47)	0.064	2.37 (0.81; 6.93)	0.117
rs34897046	C	1.16 (0.39–3.43)	0.836	0.955 (0.32–2.89)	0.934	1.04 (0.36; 3.03)	0.936
rs11931061	G	1.56 (1.05–2.33)	0.029	1.289 (0.87–1.20)	0.210	1.469 (0.99; 2.17)	0.054
rs3817444	A	1.60 (1.06–2.42)	0.026	1.117 (0.74–1.68)	0.597	1.39 (0.93; 2.08)	0.107
rs4864548	A	0.84 (0.55–1.29)	0.433	0.954 (0.63–1.46)	0.829	0.816 (0.54; 1.23)	0.335
rs726967	T	1.57 (1.03–2.37)	0.035	1.096 (0.73–1.66)	0.663	1.38 (0.92; 2.07)	0.117

* Binary logistic regression model adjusted for five first principal component analysis (PCA), sex and age; Cutoffs and sample-sizes: Inattention ≥ 9 symptoms (75 cases and 146 controls); Hyperactivity ≥ 8 symptoms (83 cases and 138 controls; global ADHD ≥ 14 symptoms (99 cases and 122 controls).

## Supplementary Materials

The following are available online at http://www.mdpi.com/2073-4425/10/2/88/s1. Table S1: Symptoms distribution in Attention Deficit Hyperactivity Disorder (ADHD) probands sample (N = 221). Table S2. Allele frequency information for European, African and American continental populations for the *CLOCK* variants explored in this study (data collected from 1000 Genomes Project Phase 3). Table S3: Linear regression results considering the number of Inattention, Hyperactivity and Total Attention Deficit Hyperactivity Disorder symptoms in probands sample (N = 221). Table S4. Single Nucleotide Polymorphism (SNP) functional information collected by online source (RegulomeDB, Haploreg v4.1, VEP and CADD v1.4). Table S5. Regulatory sites available for the eight SNPs in *CLOCK* locus in brain tissues using HaploReg v4.1. Figure S1 PCA plot using genotyped markers (pre-imputation) for the Brazilian trio samples European, American, African and Asian genotype information from 1000 Genomes Project.
