# Supplementary material for: CLOCK Polymorphisms in Attention-Deficit/Hyperactivity Disorder (ADHD): Further Evidence Linking Sleep and Circadian Disturbances and ADHD
Source: Genes (Basel). 2019 Jan 28;10(2):88. doi: 10.3390/genes10020088 (PMC6410065; doi:10.3390/genes10020088)
Supplement: Supplementary file 1 [file genes-10-00088-s001.pdf]

Table S1: Symptoms distribution in Attention Deficit Hyperactivity Disorder (ADHD) probands sample (N=221).

	Innattention Symptoms Percentiles	Hyperactivity Symptoms Percentiles	Total ADHD Symptoms Percentiles
**1%**	2	0	6
**5%**	5	0	7
**10%**	6	0	8
**25%**	7	2	9
**50%**	8	6	13
**75%**	9	8	16
**90%**	9	9	17
**95%**	9	9	18
**99%**	9	9	18
**Smallest**	0	0	6
**Largest**	10	10	18
**Nº obs.**	221	221	221
**Mean**	7.502	5.213	12.715
**Std. Dev.**	1.586	3.231	3.655
**Variance**	2.515	10.441	13.359
**Skewness**	-1.447	-0.283	-0.098
**Kurtosis**	6.185	1.581	1.710

Table S2. Allele frequency information for European, African and American continental populations for the CLOCK variants explored in this study (data collected from 1000 Genomes Project Phase 3).

SNP	EA	RA	Brazilian proband sample FREQ EA*	Europeans FREQ EA*	Africans FREQ EA*	American FREQ EA*
rs534654	A	G	0.226	0.192	0.201	0.202
rs1801260	G	A	0.253	0.305	0.152	0.238
rs6855837	T	G	0.048	0.002	0.302	0.023
rs34897046	C	G	0.029	0.045	0.004	0.024
rs11931061	G	A	0.402	0.352	0.676	0.314
rs3817444	A	C	0.344	0.350	0.232	0.284
rs4864548	A	G	0.344	0.342	0.172	0.448
rs726967	T	A	0.334	0.351	0.138	0.284

Note: EA: Effect allele, RA: Reference allele, FREQ EA: Effect (as considered in the association analysis) allele frequency.

Table S3. Linear regression results considering the number of Inattention, Hyperactivity and Total Attention Deficit Hyperactivity Disorder symptoms in probands sample (N=221).

		Inattention Symptoms				Hyperactivity Symptoms				Total ADHD Symptoms			
**SNP**	**A1**	**BETA***	**L95**	**U95**	**P**	**BETA***	**L95**	**U95**	**P**	**BETA***	**L95**	**U95**	**P**
**rs534654**	A	0.155	-0.210	0.521	0.405	0.465	-0.246	1.175	0.201	0.528	-0.320	1.375	0.224
**rs1801260**	G	- 0.037	-0.359	0.284	0.821	-0.653	-1.273	-0.033	0.040	-0.624	-1.372	0.124	0.104
**rs6855837**	T	0.134	-0.623	0.891	0.729	1.716	0.259	3.173	0.022	1.929	0.149	3.708	0.035
**rs34897046**	C	0.168	-0.663	0.999	0.692	-0.547	-2.165	1.071	0.508	-0.241	-2.174	1.692	0.807
**rs11931061**	G	0.232	- 0.059	0.529	0.119	0.718	0.156	1.279	0.013	0.943	0.268	1.619	0.007
**rs3817444**	A	0.318	0.018	0.617	0.0390	0.467	-0.119	1.053	0.120	0.744	0.050	1.441	0.038
**rs4864548**	A	-0.228	-0.541	0.084	0.154	-0.195	-0.806	0.416	0.533	-0.468	-1.195	0.260	0.209
**rs726967**	T	0.305	0.001	0.609	0.051	0.514	-0.079	1.107	0.091	0.774	0.068	1.479	0.033

*Regression adjusted for five first PCA, age and sex. Alleles considering forward strand as reference.

Table S4. **Single Nucleotide Polymorphism (SNP) functional information collected by online source (RegulomeDB, Haploreg v4.1, VEP and CADD v1.4).**

SNP ID	Location	EA	RA	Consequence	Protein position/ Aminoacids	SIFT/ PolyPhen	Regulome DB score	HaploReg Promoter histone marks	HaploReg Enhancer histone marks	HaploReg DNAse	HaploReg Proteins bound	HaploReg Motifs changed	HaploReg eQTL hits	CADD PHRED score
rs534654	56290220	G	A	3848 bp down strem the gene	-	-	5	-	BRN	-	-	E2A,GR,ZEB1	-	2.699
rs1801260	56301369	G	A	exon 23 (3'UTR)	-	-	3a	-	7 tissues	13 tissues	CTCF,BATF, STAT3	GR,ZBRK1	-	10.910
rs6855837	56319244	T	G	exon 15	395 L/I	Tolerated /benign	6	-	-	-	-	Egr-1,GATA,Irf	-	22.400
rs34897046	56325365	C	G	exon 10	208 S/C	Deleterious/benign	ND	-	8 tissues	-	-	Pou2f2,Zfp187	3 hits	24.500
rs11931061	56338793	A	G	intron 7	-	-	ND	-	-	-	-	-	6 hits	1.558
rs3817444	56375981	C	A	intron 2	-	-	5	-	9 tissues	-	-	Nanog,RXRA	9 hits	3.530
rs4864548	56413803	A	G	727 up stream the gene	-	-	1d	23 tissues	BLD, GI	16 tissues	-	YY1	43 hits	6.413
rs726967	56421713	T	A	8637 up stream the gene	-	-	6	-	-	-	-	4 altered motifs	7 hits	3.163

Functional characterization based on the transcript RefSeq NM_004898.3. Assembly GRCh37. NM_004898.3 transcript: 23 exons, 22 introns. *CLOCK* position: chr4: 56294068-56413076; ND: No Data; Alleles considering reference forward strand: EA = Effect Allele, RA= Reference allele. BRN=brain; BLD= blood, GI=gastric intestine; CTCF= Transcriptional repressor CTCF; BATF=Basic leucine zipper transcription factor; STAT3=Signal transducer and activator of transcription 3; search conducted on 18 September 2018.

Table S5. Regulatory sites available for the eight SNPs in *CLOCK locus* in brain tissues using HaploReg v4.1.

Epigenome ID (EID)	Group	Description	Chromatin states (Core 15-state model)	Chromatin states (25-state model using 12 imputed marks)	H3K4me1	H3K4me3	H3K27ac	H3K9ac	DNase
rs534654									
E071	Brain	Brain Hippocampus Middle	6_EnhG	-	H3K4me1_Enh	-	H3K27ac_Enh	-	-
E074	Brain	Brain Substantia Nigra	-	-	H3K4me1_Enh	-	H3K27ac_Enh	-	-
E068	Brain	Brain Anterior Caudate	-	-	H3K4me1_Enh	-	H3K27ac_Enh	H3K9ac_Pro	-
E069	Brain	Brain Cingulate Gyrus	6_EnhG	-	H3K4me1_Enh	-	H3K27ac_Enh	-	-
E072	Brain	Brain Inferior Temporal Lobe	-	-	H3K4me1_Enh	-	H3K27ac_Enh	-	-
E067	Brain	Brain Angular Gyrus	6_EnhG	-	H3K4me1_Enh	-	H3K27ac_Enh	-	-
E073	Brain	Brain_Dorsolateral_Prefrontal_Cortex	-	-	H3K4me1_Enh	-	H3K27ac_Enh	-	-
E070	Brain	Brain Germinal Matrix	-	-		-		-	-
E082	Brain	Fetal Brain Female	-	-	-	-	-	-	-
E081	Brain	Fetal Brain Male	-	-	-	-	-	-	-
rs1801260									
E071	Brain	Brain Hippocampus Middle	-	11_TxEnh3	H3K4me1_Enh		H3K27ac_Enh	-	-
E074	Brain	Brain Substantia Nigra	-	11_TxEnh3	H3K4me1_Enh		H3K27ac_Enh	-	-
E068	Brain	Brain Anterior Caudate	-	11_TxEnh3	H3K4me1_Enh		H3K27ac_Enh	H3K9ac_Pro	-
E069	Brain	Brain Cingulate Gyrus	-	11_TxEnh3	H3K4me1_Enh		H3K27ac_Enh	-	-
E072	Brain	Brain Inferior Temporal Lobe	-	11_TxEnh3	H3K4me1_Enh		H3K27ac_Enh	H3K9ac_Pro	-
E067	Brain	Brain Angular Gyrus	-	11_TxEnh3	H3K4me1_Enh		H3K27ac_Enh	H3K9ac_Pro	-
E073	Brain	Brain_Dorsolateral_Prefrontal_Cortex	6_EnhG	11_TxEnh3	H3K4me1_Enh		H3K27ac_Enh	-	-
E070	Brain	Brain Germinal Matrix	-	-	-	-	-	-	-
E082	Brain	Fetal Brain Female	-	-	-	-	-	-	-
E081	Brain	Fetal Brain Male	-	-	-	-	-	-	-

rs6855837									
E071	Brain	Brain Hippocampus Middle	-	-	-	-	-	-	-
E074	Brain	Brain Substantia Nigra	-	-	-	-	-	-	-
E068	Brain	Brain Anterior Caudate	-	-	-	-	-	H3K9ac_Pro	-
E069	Brain	Brain Cingulate Gyrus	-	-	-	-	H3K27ac_Enh	-	-
E072	Brain	Brain Inferior Temporal Lobe	-	-	-	-	-	-	-
E067	Brain	Brain Angular Gyrus	-	-	-	-	-	-	-
E073	Brain	Brain_Dorsolateral_Prefrontal_Cortex	-	-	-	-	-	-	-
E070	Brain	Brain Germinal Matrix	-	-	-	-	-	-	-
E082	Brain	Fetal Brain Female	-	-	-	-	-	-	-
E081	Brain	Fetal Brain Male	-	-	-	-	-	-	-
rs34897046									
E071	Brain	Brain Hippocampus Middle	-	-	-	-	-	-	
E074	Brain	Brain Substantia Nigra	-	-	-	-	-	-	
E068	Brain	Brain Anterior Caudate	-	-	-	-	-	-	
E069	Brain	Brain Cingulate Gyrus	-	-	-	-	-	-	
E072	Brain	Brain Inferior Temporal Lobe	-	-	-	-	-	-	
E067	Brain	Brain Angular Gyrus	-	-	-	-	-	-	
E073	Brain	Brain_Dorsolateral_Prefrontal_Cortex	-	-	-	-	-	-	
E070	Brain	Brain Germinal Matrix	-	-	-	-	-	-	
E082	Brain	Fetal Brain Female	-	-	-	-	-	-	
E081	Brain	Fetal Brain Male	-	-	-	-	-	-	
rs11931061									
E071	Brain	Brain Hippocampus Middle	-	-	-	-	-	-	
E074	Brain	Brain Substantia Nigra	-	-	-	-	-	-	
E068	Brain	Brain Anterior Caudate	-	-	-	-	-	-	
E069	Brain	Brain Cingulate Gyrus	-	-	-	-	-	-	
E072	Brain	Brain Inferior Temporal Lobe	-	-	-	-	-	-	

E067	Brain	Brain Angular Gyrus	-	-	-	-	-	-	
E073	Brain	Brain_Dorsolateral_Prefrontal_Cortex	-	-	-	-	-	-	
E070	Brain	Brain Germinal Matrix	-	-	-	-	-	-	
E082	Brain	Fetal Brain Female	-	-	-	-	-	-	
E081	Brain	Fetal Brain Male	-	-	-	-	-	-	
rs3817444									
E071	Brain	Brain Hippocampus Middle	-	-	-	-	-	-	
E074	Brain	Brain Substantia Nigra	-	-	-	-	-	-	
E068	Brain	Brain Anterior Caudate	-	-	-	-	-	-	
E069	Brain	Brain Cingulate Gyrus	-	-	-	-	-	-	
E072	Brain	Brain Inferior Temporal Lobe	-	-	-	-	-	-	
E067	Brain	Brain Angular Gyrus	-	-	-	-	-	-	
E073	Brain	Brain_Dorsolateral_Prefrontal_Cortex	-	-	-	-	-	-	
E070	Brain	Brain Germinal Matrix	-	-	-	-	-	-	
E082	Brain	Fetal Brain Female	-	-	-	-	-	-	
E081	Brain	Fetal Brain Male	-	-	-	-	-	-	
rs4864548									
E071	Brain	Brain Hippocampus Middle	2_TssAFlnk	2_PromU	H3K4me1_Enh	H3K4me3_Pro	H3K27ac_Enh	-	-
E074	Brain	Brain Substantia Nigra	2_TssAFlnk	2_PromU	H3K4me1_Enh	H3K4me3_Pro	H3K27ac_Enh	H3K9ac_Pro	-
E068	Brain	Brain Anterior Caudate	2_TssAFlnk	2_PromU	-	H3K4me3_Pro	H3K27ac_Enh	H3K9ac_Pro	-
E069	Brain	Brain Cingulate Gyrus	2_TssAFlnk	2_PromU	H3K4me1_Enh	H3K4me3_Pro	H3K27ac_Enh	H3K9ac_Pro	-
E072	Brain	Brain Inferior Temporal Lobe	2_TssAFlnk	2_PromU	H3K4me1_Enh	H3K4me3_Pro	H3K27ac_Enh	H3K9ac_Pro	-
E067	Brain	Brain Angular Gyrus	2_TssAFlnk	2_PromU	H3K4me1_Enh	H3K4me3_Pro	H3K27ac_Enh	H3K9ac_Pro	-
E073	Brain	Brain_Dorsolateral_Prefrontal_Cortex	1_TssA	2_PromU	H3K4me1_Enh	H3K4me3_Pro	H3K27ac_Enh	H3K9ac_Pro	-
E070	Brain	Brain Germinal Matrix	1_TssA	2_PromU	-	H3K4me3_Pro	-	-	-
E082	Brain	Fetal Brain Female	1_TssA	2_PromU	H3K4me1_Enh	H3K4me3_Pro	-	-	DNase
E081	Brain	Fetal Brain Male	1_TssA	2_PromU	H3K4me1_Enh	H3K4me3_Pro	-	-	-
rs726967									

E071	Brain	Brain Hippocampus Middle	-	-	-	-	-	-	-
E074	Brain	Brain Substantia Nigra	-	-	-	-	-	-	-
E068	Brain	Brain Anterior Caudate	-	-	-	-	-	H3K9ac_Pro	-
E069	Brain	Brain Cingulate Gyrus	-	-	-	-	-	-	-
E072	Brain	Brain Inferior Temporal Lobe	-	-	-	-	-	H3K9ac_Pro	-
E067	Brain	Brain Angular Gyrus	-	-	-	-	-	-	-
E073	Brain	Brain_Dorsolateral_Prefrontal_Cortex	-	-	-	-	-	-	-
E070	Brain	Brain Germinal Matrix	-	-	-	-	-	-	-
E082	Brain	Fetal Brain Female	-	-	-	-	-	-	-
E081	Brain	Fetal Brain Male	-	-	-	-	-	-	-

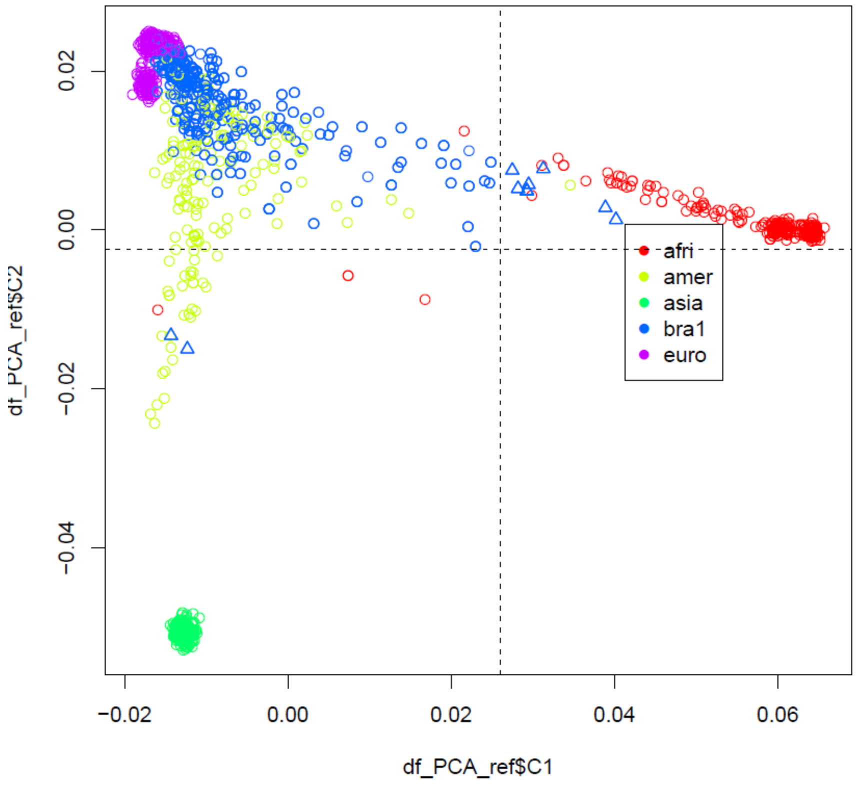

Figure S1: PCA plot using genotyped markers (pre-imputation) for the Brazilian trio samples European, American, African and Asian genotype information from 1000 Genomes Project. Triangles denote outliers excluded before the imputation process and statistical analyses. The dashed lines denote the 3-standard deviation from the mean PC variable (considering Brazilian sample).
